# Investigating the Added Value of FreeSurfer’s Manual Editing Procedure for the Study of the Reading Network in a Pediatric Population

**DOI:** 10.3389/fnhum.2020.00143

**Published:** 2020-04-24

**Authors:** Caroline Beelen, Thanh Vân Phan, Jan Wouters, Pol Ghesquière, Maaike Vandermosten

**Affiliations:** ^1^Parenting and Special Education Research Unit, Faculty of Psychology and Educational Sciences, KU Leuven, Leuven, Belgium; ^2^Icometrix Company, Leuven, Belgium; ^3^Research Group ExpORL, Department of Neuroscience, KU Leuven, Leuven, Belgium

**Keywords:** FreeSurfer, manual editing, automated processing, pediatric T1-weighted images, reading network, developmental neuroimaging

## Abstract

Insights into brain anatomy are important for the early detection of neurodevelopmental disorders, such as dyslexia. FreeSurfer is one of the most frequently applied automatized software tools to study brain morphology. However, quality control of the outcomes provided by FreeSurfer is often ignored and could lead to wrong statistical inferences. Additional manual editing of the data may be a solution, although not without a cost in time and resources. Past research in adults on comparing the automatized method of FreeSurfer with and without additional manual editing indicated that although editing may lead to significant differences in morphological measures between the methods in some regions, it does not substantially change the sensitivity to detect clinical differences. Given that automated approaches are more likely to fail in pediatric—and inherently more noisy—data, we investigated in the current study whether FreeSurfer can be applied fully automatically or additional manual edits of T1-images are needed in a pediatric sample. Specifically, cortical thickness and surface area measures with and without additional manual edits were compared in six regions of interest (ROIs) of the reading network in 5-to-6-year-old children with and without dyslexia. Results revealed that additional editing leads to statistical differences in the morphological measures, but that these differences are consistent across subjects and that the sensitivity to reveal statistical differences in the morphological measures between children with and without dyslexia is not affected, even though conclusions of marginally significant findings can differ depending on the method used. Thereby, our results indicate that additional manual editing of reading-related regions in FreeSurfer has limited gain for pediatric samples.

## Introduction

The anatomy of the brain is considered to play a major role in neurodevelopmental disorders, and investigating anatomical features of the brain at pre-diagnostic age could offer us insights into the causal brain mechanisms and early neural markers of these disorders. Consequentially, it allows us to apply early intervention tools, which have demonstrated to be effective (Jones et al., 2007; Makrygianni and Reed, 2010; Peters-Scheffer et al., 2011; Estes et al., 2015; Lovett et al., 2017). In the case of dyslexia, starting intervention at the end of kindergarten has shown to be most efficient for closing the gap in reading performances with typically developing peers (Wanzek and Vaughn, 2007; Ozernov-Palchik and Gaab, 2016). Anatomical features of the brain can be studied non-invasively by using structural MRI (T1) scans, and this can lead to a better understanding of behavioral characteristics of developmental disorders, such as dyslexia (Durston et al., 2001). One of the most frequently applied software packages for structural brain imaging analysis world-wide is FreeSurfer, an open-access software program for the automated surface-based reconstruction of brain images (Dale et al., 1999; Fischl et al., 1999a). In the current study, we will examine whether FreeSurfer can be applied in a fully automated way or whether additional manual edits are needed to improve the automated analysis when studying regions of the reading network in a pediatric population of 5-to-6-year-old children with and without dyslexia.

FreeSurfer has been developed by the Martino Center for Biomedical Imaging with the purpose to be robust, accurate and easy to use (Fischl et al., 2002, 2004). The software tool has an automatic reconstruction pipeline for the processing of anatomical brain images, which involves several processing steps. The first step is skull stripping, motion artifact correction, and B1 bias field correction. The second step is gray-white matter segmentation based on a deformable surface template defined in MNI305 space. As an alternative, a template can be created from one’s study sample (i.e., the average subject will form the template) with the advantage of the optimal formation of white and pial surfaces. The third step is region labeling on the cortical surface that is performed by non-linear registration of the cortical surface of the subject with the Desikan-Killiany/Destrieux atlas (Desikan et al., 2006; Destrieux et al., 2010). FreeSurfer adopts a probabilistic approach based on Markov random fields for automated labeling of brain regions by the implementation of a brain surface atlas that is generated by a training set of 40 manually labeled brains, created from 10 young adults (mean age = 21.5, age-range 19–24; six females, four males), 10 middle-aged adults (mean age = 49.8, age-range 41–57; seven females, three males), 10 typically developing elderly (mean age = 74.3, age-range 66–87; eight females, two males) and 10 elderly with Alzheimer’s disease (mean age = 78.2, age-range 71–86; five females, five males; Desikan et al., 2006; Makowski et al., 2018). Outcome measures of the morphological analyses are, amongst other, surface area, cortical thickness and volume (Dale et al., 1999; Fischl et al., 1999a,b, 2001; Fischl and Dale, 2000). *The surface area* corresponds to the white surface (gray-white matter boundary), *cortical thickness* corresponds to the distance between the white surface and the pial surface [gray matter-cerebrospinal fluid (CSF) boundary], and *volume* is the product of surface area and cortical thickness (Fischl and Dale, 2000). It is suggested that surface area and cortical thickness offer independent, but complementary information on brain anatomy, since they have different genetic sources (Panizzon et al., 2009; Winkler et al., 2010) and follow different developmental trajectories (Kapellou et al., 2006).

Over the past few decades, automated methods have increased in popularity for analyzing brain morphology because of increased efficiency (i.e., reduced time and costs) of the analyses (Eickhoff et al., 2015). Although past research indicated that cortical thickness obtained by the automated processing stream of FreeSurfer has good agreement with cortical thickness from histological and manual measurements (Fischl et al., 2002; Rosas et al., 2002; Cardinale et al., 2014), FreeSurfer recommends^1^ to always visually check and, if observed necessary, manually adapt images in between the automatic processing stages, thereby optimizing the parcellation and segmentation of the brain images. The automatic reconstruction process can be interrupted for manual adjustments after specific processing stages, including skull stripping, intensity normalization, white matter segmentation and surface extraction (see Figure 1 of the Supplementary Information). Manual adjustments include fixing skull stripping errors, intensity normalization errors, topological errors, white matter errors, and pial errors (see the Supplementary Information). They might be necessary when an experimenter visually observes that the image is not well parcellated or segmented, as indicated by too little or too much skull of the brain left behind after skull stripping, incorrect placements of the pial surface or white surface, and the presence of small holes on the brain surface (visible on the inflated brain image). However, manually adapting images has disadvantages. First and foremost, it is a very time-consuming, labor-intensive task. In their guidelines, FreeSurfer suggests to take around 30 min per image to fix errors, yet in reality, such time window seems far too short as per image two-hundred slices, preferably in four different views (i.e., coronal, sagittal, axial and 3D-vision), need to be visually checked and possibly edited using five different fixing steps (for details on the editing process see the Supplementary Information). Also, manual editing requires a certain level of expertise, which can only be gained through practice and trial-and-error (Canna et al., 2018). The experimenter needs to get experience with how many edits at which locations are needed to obtain a desired change. To visualize the outcomes of the editing process, the image first has to undergo the automatic reconstruction process again. Another disadvantage of manual editing is that compared to the automated reconstruction of brain images, manual editing is more prone to inter-subject variation and rater drift (i.e., rater variation over time; Fischl et al., 2002; White et al., 2018). Especially in large databases, in which data are segmented over a prolonged period, intra-rater reliability will be difficult to maintain as rater drift becomes more significant over time (Spinks et al., 2002; Nugent et al., 2007). Due to the absence of a standardized detailed protocol on performing manual adjustments in FreeSurfer, inter-subject variation and rater drift might be considerably larger after manually editing the data.

FreeSurfer’s fully automated approach and the automated approach with additional manual edits have been compared in former adult studies, more specifically in healthy adults (Canna et al., 2018; Waters et al., 2019), adults with a history of severe head injuries (Guenette et al., 2018) and in adults with 22q11.2 deletion syndrome (McCarthy et al., 2015). Each of these studies showed using paired samples *t*-tests that both methods did not significantly differ in their regions of interest (ROIs; McCarthy et al., 2015) or only in a minority of their ROIs (Canna et al., 2018; Waters et al., 2019). Furthermore, the intra-class correlations measuring consistency in differences between the methods across subjects were high for the ROIs included in these studies (McCarthy et al., 2015; Guenette et al., 2018; Waters et al., 2019), except for a few subcortical ROIs (Guenette et al., 2018). Finally, these studies showed that the automated method with additional manual edits does not have an increased sensitivity to detect differences when comparing a clinical group (McCarthy et al., 2015) or investigating individual brain-behavior relationships (Waters et al., 2019) between the methods. Hence, according to these studies, manual editing does not lead to differences in absolute values between the methods in most ROIs. Additionally, differences between the methods— in situations in which they occurred—are consistent, and manual editing does not lead to an increased sensitivity to detect individual or clinical group differences. However, since these studies were conducted in adults, their outcomes might not apply to pediatric populations. Manual adaptations are needed to a considerably larger extent in pediatric data, since non-linear and region-specific brain development leads to significant differences between adult and child brains (Phan et al., 2018b). In pediatric populations, segmentation and registration of MRI data is more prone to errors because of the use of an adult template (Muzik et al., 2000; Yoon et al., 2009; Phan et al., 2018a). Also, MRI images of pediatric populations are generally noisier and more prone to artifacts due to in-scanner motion that is higher in children (Brown et al., 2010; van Dijk et al., 2012). Head motion, in particular, will lead to severe ringing, blurring, and ghosting artifacts, hindering the determination of tissue boundaries, thereby risking the production of invalid outcome measures (Backhausen et al., 2016; Phan et al., 2018a, b). Even subtle motion, not easily detected by visual inspection, will lead to systematic biases in the automatic measurements of structural brain properties (Blumenthal et al., 2002; Reuter et al., 2015; Alexander-Bloch et al., 2016). Errors are comparable to yearly atrophy rates in neurodegenerative diseases (Barkhof et al., 2009; Rosas et al., 2011) or yearly growth rates of normal developing brain tissues (Hedman et al., 2012). Influences of motion on outcomes generated by the automated image processing pipeline of FreeSurfer (Blumenthal et al., 2002; Reuter et al., 2015) are significant; in adults, a small increase in motion led to 1.4%–2.0% gray matter volume reduction (Reuter et al., 2015), and in children, a small amount of motion led to 4% gray matter volume reduction, a moderate amount to 7% gray matter volume reduction and a large amount to 27% gray matter volume reduction (Blumenthal et al., 2002). Despite these difficulties faced with implementing FreeSurfer’s automated pipeline in pediatric samples, over the past few years there has been an increased use of FreeSurfer in pediatric samples, resulting from an increased interest in early-onset identification of neurodevelopmental disorders (Merkley et al., 2008; Wolosin et al., 2009; Fallucca et al., 2011; Widjaja et al., 2011; Overvliet et al., 2013; Wozniak et al., 2013; Mayer et al., 2015; Mahajan et al., 2016; Yang et al., 2016; Gold et al., 2017). Only a few of these studies mentioned whether they had performed manual adaptations (Merkley et al., 2008; Fallucca et al., 2011; Gold et al., 2017), and only one mentioned the actual editing process by briefly informing which type of edits at which locations had been made (Merkley et al., 2008). Currently, little is known about the influence of additional manual editing pediatric samples in FreeSurfer on morphological outcome measures.

In the current study, we will assess the added value of FreeSurfer’s manual editing tool on outcome values of surface area and cortical thickness that are generated by FreeSurfer’s automated reconstruction pipeline in a pediatric population of 5-to-6-year-old children with and without dyslexia. We take a similar approach as McCarthy et al. (2015) who investigated this in adults. First, we will investigate whether outcome measures obtained with the automated vs. semi-automated (i.e., additional manually edited data) method differ significantly, and whether these differences are consistent across subjects. We restrict our analyses to six pre-defined ROIs of the Desikan-Killiany atlas that belong to the reading network (Richlan et al., 2009; Beelen et al., 2019); the fusiform gyrus, the inferior parietal gyrus, the inferior temporal gyrus, the middle temporal gyrus, the pars opercularis of the inferior frontal gyrus and the superior temporal gyrus, as well as their right homologous counterparts. Then, we will examine whether the implementation of the fully automated approach (with no manual edits) affects the sensitivity to find differences between groups (in our case pre-readers with and without dyslexia diagnosis) by analyzing the difference in effect size between the fully automated method and the automated method with additional edits for the outcome measures surface area and cortical thickness. In our pediatric study, it is expected that the methods will significantly differ from one another, since pediatric data as opposed to adult data require more manual adjustments due to a non-matching template and increased in-scanner head motion. In our former pediatric study (Beelen et al., 2019), we observed for the automated method with manual edits significant group differences in the surface area of the bilateral fusiform gyrus between children with and without dyslexia. However, a comparison with the fully automated approach was not made. Hence, it remains to be seen whether manually editing pediatric images in FreeSurfer will result into an increased sensitivity to detect statistical differences between children with and without dyslexia.

## Materials and Methods

### Participants

Participants were 54 Flemish children, of whom 31 children with (FRD^+^) and 23 children without (FRD^−^) a family risk for dyslexia, defined as a first-degree relative (parent or sibling) with a clinical diagnosis of dyslexia. This sample is part of a larger longitudinal project (Vanvooren et al., 2014), of which the current sample is identical to the one described in Beelen et al. (2019). Participants underwent cognitive-behavioral tests once a year and EEG and MRI sessions alternately once every 2 years from the last year of kindergarten until the 5th grade of primary school. For the first MRI session, participants were trained with a child-friendly “submarine” protocol (Theys et al., 2014). This protocol was invented to make the participants familiar and at ease with the scanning procedure to reduce in-scanner motion artifacts. Originally, 71 participants underwent the first MRI session, but due to excessive motion in the scanner 17 participants were excluded from the study, resulting in the remaining sample of 54 participants. Images of the excluded participants had severe blurring, ringing or ghosting artifacts due to which they were unusable for analysis purposes according to the Blumenthal criteria (Blumenthal et al., 2002; Phan et al., 2018a). Images of the 54 participants that were included in the study had no, mild or moderate ringing, blurring or ghosting artifacts.

The cognitive-behavioral tests that were obtained from the participants from grade 2 onwards each year included a standardized word reading (Brus and Voeten, 1973) and pseudo-word reading (van den Bos et al., 1994) test. Reading tests were administered during the first semester of the 2nd to 4th grade, and the second semester of the 5th grade of primary school. Based on these tests, participants were retrospectively classified as typical readers (TR, *n* = 38) or readers with dyslexia (DR, *n* = 16). Children scoring below the 10th percentile on either the word reading or pseudo-word reading test at the three last time points were classified as readers with dyslexia (*n* = 15). If reading had only been assessed at two-time points, the child’s reading score had to be below the 10th percentile at both time points on either the word reading or pseudo-word reading test to be classified as a reader with dyslexia (*n* = 1). In our study, 45% of FRD^+^ children (*n* = 14) and 9% of FRD^−^ children (*n* = 2) fulfilled our dyslexia criteria (*n* = 16). Demographics and behavioral assessment scores of the study sample were mentioned in our former study (Beelen et al., 2019). The study was approved by the local ethical committee of the university hospital (UZ Leuven) and following ethical standards described within the declaration of Helsinki. The study has not been pre-registered. Informed consent had been obtained from the parents.

### Image Acquisition

Scanning sessions of participants took place at the university hospital of Leuven (UZ Leuven). Total scanning time was nearly half an hour and T1-weighted brain images were acquired within 6 min. and 22 s. Scans were taken with a Philips 3T-scanner (Best, Netherlands) with 3D Turbo field echo and a 32-channel head coil. Per the scanning session, 182 contiguous coronal slices were collected with the following parameter settings: TR = 9.6 ms; TE = 4.6 ms; flip angle = 8°; FOV = 250 × 250 × 218 mm^3^; voxel size = 1 × 1 × 1.2 mm^3^.

### Processing of T1-Weighted Images

The T1-weighted images were processed by the automated cross-sectional reconstruction processing stream in FreeSurfer version 5.3 on a Linux Ubuntu software system version 14.02. First, an isotropic brain image was created with all non-brain tissue removed by using a hybrid watershed/surface deformation procedure (Ségonne et al., 2004). Second, motion correction and b1-bias field correction were applied. Furthermore, images underwent segmentation of gray/white matter structures (Fischl et al., 2002, 2004), intensity normalization (Sied et al., 1998), gray/white matter boundary tessellation, automated topological correction (Fischl et al., 2001; Ségonne et al., 2007) and surface deformation following intensity gradients to optimally place gray matter/white matter/CSF borders (Dale and Sereno, 1993; Dale et al., 1999; Fischl and Dale, 2000). Thereafter, the Desikan-Killiany atlas was used to perform the cortical parcellation. The atlas automatically subdivides the human cortex into 34 gyral regions-of-interest based on anatomical markers of curvature and sulcal information on the inflated brain images (Desikan et al., 2006). For our study, we selected six ROIs that belonged to the reading network and their right homologous counterparts from the Desikan-Killiany atlas, which were bilaterally the fusiform gyrus, the inferior parietal gyrus, the inferior temporal gyrus, the middle temporal gyrus, the pars opercularis of the inferior frontal gyrus and the superior temporal gyrus (Beelen et al., 2019). Finally, signal-to-noise ratio (SNR) was calculated using the following formula (see Gedamu et al., 2008, p. 313):

$$\text{SNR}=\frac{\mu_{\text{signal intensity}}}{0.8\cdot \sigma_{\text{noise intensity}}}$$

*“… where signal intensity is the mean of the signal intensity distribution and noise intensity is the standard deviation of the noise intensity distribution of the image. Dividing by a factor of 0.8 is done to compensate for the Rayleigh distribution effect in the background noise.”* Mean signal intensity corresponds to the mean intensity of white matter. In our sample, mean SNR = 977.36 dB; SD = 3324.54 dB.

### Manual Intervention

The data that underwent the automated cross-sectional processing stream were copied. The copies underwent an additional manual editing procedure (i.e., automated data with additional edits), whereas the original processed images did not (i.e., fully automated data). The reconstructed brain images that underwent additional manual editing were adjusted in FreeSurfer’s supporting toolbox Freeview. Manual edits were performed according to a self-written manual editing protocol (see the Supplementary Information for a broad description of the editing process). In short, editing consisted of the following steps: (1) fixing skull stripping errors; (2) fixing intensity normalization errors; (3) fixing topological errors; (4) fixing white matter errors; and (5) fixing pial errors. After the manual adjustments were completed, the images underwent the automated reconstruction process again and were visually checked for remaining errors. If necessary, the same process was repeated.

### Statistical Analyses

Statistical analyses were performed in IBM SPSS, version 25.0 (IBM Corp. 2017). Full factorial mixed-effect model analyses were run to compare surface area and cortical thickness of the ROIs between edited and unedited data. As a starting point, the following two models were tested: surface area/cortical thickness = method (2) + region (6) + hemisphere (2) + method*region (12) + method*hemisphere (12) + region*hemisphere (12) + method*region*hemisphere (24), and by using a backward selection procedure, the best models (i.e., with the lowest AIC) were selected for both outcome measures. The selected models were: surface area/cortical thickness = method (2) + region (6) + hemisphere (2) + region*hemisphere (12). All independent factors in the model were fixed, and the subject was the random intercept. Uncorrected results were presented. In the next step, we investigated the consistency of the outcome measures surface area and cortical thickness between the methods by calculating intra-class correlations (ICCs) per ROI and across subjects using a two-way mixed effect model. In a final step, to analyze whether manual edits increase the sensitivity to find statistical group differences between children with (DR) and without (TR) dyslexia, the *p*-value and effect size (Hedges’ *g*) of the mean difference in cortical thickness and surface area between DR and TR were calculated per ROI for each method, and the effect sizes were compared across ROIs between the methods using paired samples *t-tests*.

## Results

**Figure 1:**
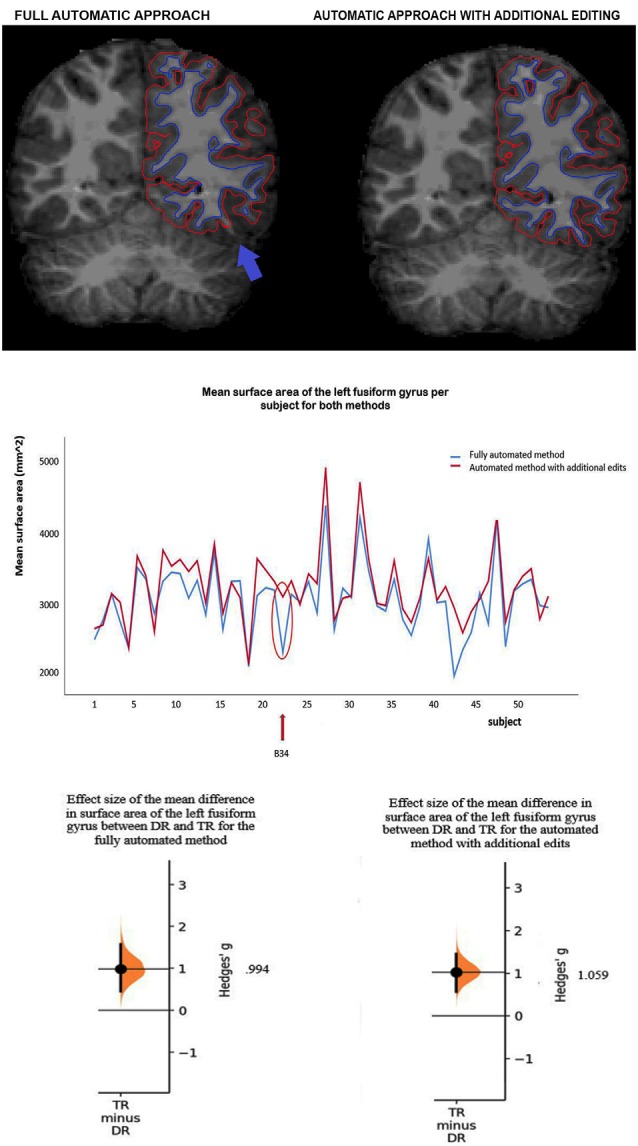
**(A)** Example of a subject (B34) with a clear difference in mean surface area of the left fusiform gyrus between the methods. Red lines represent the pial surface [i.e., the border between gray matter and cerebrospinal fluid (CSF)] and dark blue lines the white surface (i.e., the border between white matter and gray matter, the surface area). The subject has a lower mean surface area of the left fusiform gyrus (see the blue arrow) after full-automatic processing (left) as opposed to after additional manual editing (right). **(B)** The mean surface area of the left fusiform gyrus per subject for the fully automated method and the automated method with additional edits. The intra-class correlation (ICC) corresponds to the difference in surface area between the methods across subjects (ICC value = 0.939, see Table 2A). The red arrow and circle indicate for both methods the mean surface area of the left fusiform gyrus for subject B34. **(C)** Effect sizes (Hedges g’) of the mean difference in surface area of the left fusiform gyrus between children with (DR) and without (TR) dyslexia for the fully automated method (*g* = 0.994) and the automated method with additional edits (*g* = 1.059).

Method comparisons were performed across ROIs and subjects (DR and TR), and ICCs were calculated per ROI and across subjects. Furthermore, sensitivity between the methods was tested by comparing the size of the group differences between DR and TR. For surface area, results revealed that there is a significant difference between the methods (*F*_(1,1242)_ = 75.67; *p* < 0.001). The automated method with additional edits (μ = 3,229.1 mm^2^; *SE* = 45.7 mm^2^) has overall a significantly higher mean surface area than the fully automated method (μ = 3,077.0 mm^2^; *SE* = 45.7 mm^2^; for an example of the difference in surface area between the methods in an individual subject, see Figure 1A). Also, there are no interaction effects between method and ROI or method, hemisphere and ROI, which suggests that results are similar across ROIs. Table 1A indicates that for each ROI the mean surface area is larger for the automated method with additional edits than the fully automated method. For cortical thickness, results also revealed that there is a significant difference between the methods (*F*_(1,1242)_ = 7.03; *p* = 0.008). The automated method with additional edits (μ = 2,890.9 μm; *SE* = 25.8 μm) has overall a significantly lower mean thickness than the fully automated method (μ = 2,914.5 μm; *SE* = 25.8 μm). Also, there are no interaction effects between method and ROI or method, hemisphere and ROI, which suggests that results are similar across ROIs. Table 1B indicates that for each ROI the mean cortical thickness is smaller for the automated method with additional edits than the fully automated method. For surface area and cortical thickness, ICC values indicated for each ROI excellent consistency between the methods (ICCs > 0.90; see Table 2A,B and Figure 1B). Finally, the effect sizes measuring mean differences between DR and TR across ROIs were not significantly different between the methods for surface area (*t*_(11)_ = −1.72; *p* = 0.113) or cortical thickness *t*_(11)_ = 2.04; *p* = 0.066). Significant group differences between DR and TR were observed in the surface area of the bilateral fusiform gyrus for both the automated data with additional edits (see also Beelen et al., 2019) and the fully automated data (see 3A). Also, the accompanying effect sizes were large (see Table 3A and Figure 1C). Contrary, border significant results were observed in the surface area of the right inferior temporal gyrus for the automated method with additional edits as opposed to the fully automated method (Table 3A), and in the cortical thickness of the pars opercularis of the left inferior frontal gyrus for the fully automated method as opposed to the automated method with additional edits (Table 3B). Hence, depending on the choice of method border significant results lead to different conclusions on group differences between DR and TR.

**Table 1:** For both methods per ROI the mean and standard deviation.

ROI	Mean and SD for the fully automated method	Mean and SD for the automated method with additional manual edits
**(A)**		
Fusiform g. left	*M* = 3,090.93 *SD* = 485.47	*M* = 3,256.26 *SD* = 498.68
Fusiform g. right	*M* = 2,953.93 *SD* = 409.25	*M* = 3,129.02 *SD* = 447.76
Inferior parietal g. left	*M* = 4,276.39 *SD* = 532.61	*M* = 4,467.59 *SD* = 546.43
Inferior parietal g. right	*M* = 5,145.48 *SD* = 712.30	*M* = 5,338.54 *SD* = 729.91
Inferior temporal g. left	*M* = 2,800.80 *SD* = 419.59	*M* = 3,008.20 *SD* = 411.68
Inferior temporal g. right	*M* = 2,780.28 *SD* = 434.67	*M* = 2,977.48 *SD* = 431.43
Middle temporal g. left	*M* = 2,768.91 *SD* = 476.36	*M* = 2,913.91 *SD* = 470.51
Middle temporal g. right	*M* = 3,201.00 *SD* = 439.69	*M* = 3,351.52 *SD* = 431.69
Pars opercularis of the inferior frontal g. left	*M* = 1,532.00 *SD* = 261.91	*M* = 1,603.46 *SD* = 323.45
Pars opercularis of the inferior frontal g. right	*M* = 1,311.87 *SD* = 215.36	*M* = 1,349.24 *SD* = 211.44
Superior temporal g. left	*M* = 3,620.76 *SD* = 448.58	*M* = 3,781.39 *SD* = 410.60
Superior temporal g. right	*M* = 3,441.19 *SD* = 408.07	*M* = 3,572.22 *SD* = 378.57
**(B)**		
Fusiform g. left	*M* = 2,980.83 *SD* = 207.23	*M* = 2,939.43 *SD* = 190.28
Fusiform g. right	*M* = 3,094.72 *SD* = 202.26	*M* = 3,053.04 *SD* = 167.55
Inferior parietal g. left	*M* = 2,492.09 *SD* = 264.42	*M* = 2,466.02 *SD* = 263.78
Inferior parietal g. right	*M* = 2,631.57 *SD* = 256.81	*M* = 2,610.78 *SD* = 232.49
Inferior temporal g. left	*M* = 2,792.69 *SD* = 253.34	*M* = 2,774.57 *SD* = 265.17
Inferior temporal g. right	*M* = 2,912.50 *SD* = 269.33	*M* = 2,895.56 *SD* = 216.24
Middle temporal g. left	*M* = 2,975.56 *SD* = 330.09	*M* = 2,962.93 *SD* = 333.42
Middle temporal g. right	*M* = 3,105.20 *SD* = 308.95	*M* = 3,100.63 *SD* = 242.42
Pars opercularis of the inferior frontal g. left	*M* = 2,949.63 *SD* = 223.13	*M* = 2,912.61 *SD* = 240.76
Pars opercularis of the inferior frontal g. right	*M* = 2,860.28 *SD* = 241.91	*M* = 2,823.09 *SD* = 237.54
Superior temporal g. left	*M* = 3,037.09 *SD* = 241.35	*M* = 3,028.31 *SD* = 230.00
Superior temporal g. right	*M* = 3,142.09 *SD* = 214.90	*M* = 3,123.39 *SD* = 195.58

*(A) For surface area, for each method per ROI and per hemisphere the mean and standard deviation (SD) in mm^2^. (B) For cortical thickness, for each method per ROI and per hemisphere the mean and standard deviation (SD) in μm*.

**Table 2:** Intra-class correlations (ICCs) per ROI and across subjects.

ROI	ICCs for the left hemisphere	ICCs for the right hemisphere
**(A)**		
Fusiform g.	0.939	0.941
Inferior parietal g.	0.944	0.959
Inferior temporal g.	0.911	0.943
Middle temporal g.	0.970	0.963
Pars opercularis of the inferior frontal g.	0.950	0.957
Superior temporal g.	0.962	0.965
**(B)**		
Fusiform g.	0.938	0.928
Inferior parietal g.	0.935	0.955
Inferior temporal g.	0.951	0.941
Middle temporal g.	0.966	0.944
Pars opercularis of the inferior frontal g.	0.968	0.948
Superior temporal g.	0.959	0.960

*(A) For surface area, for both hemispheres per ROI and across subjects the intra-class correlations (ICCs) between the methods based on a two-way mixed-effects model. (B) For cortical thickness, for both hemispheres per ROI and across subjects the intra-class correlations (ICCs) between the methods based on a two-way mixed-effects model*.

**Table 3:** For both methods per ROI the *P*-values and effect sizes (Hedges’ *g*) of reading group differences (DR vs. TR.)

ROI	*P*-values and effect sizes (Hedges’ *g*) of reading group differences (DR vs. TR) for the fully automated method	*P*-values and effect sizes (Hedges’ *g*) of reading group differences (DR vs. TR) for the automated method with additional manual edits
**(A)**		
Fusiform g. left	*t*_(52)_ = 3.34; *p* = 0.002; *g* = 0.994	*t*_(52)_ = 3.55; *p* = 0.001; *g* = 1.059
Fusiform g. right	*t*_(52)_ = 2.83; *p* = 0.007; *g* = 0.842	*t*_(52)_ = 2.96; *p* = 0.005; *g* = 0.879
Inferior parietal g. left	*t*_(52)_ = −0.43; *p* = 0.671; *g* = 0.129	*t*_(52)_ = −0.49; *p* = 0.628; *g* = 0.145
Inferior parietal g. right	*t*_(52)_ = 0.01; *p* = 0.993; *g* = 0.003	*t*_(52)_ = 0.44; *p* = 0.663; *g* = 0.131
Inferior temporal g. left	*t*_(52)_ = 0.98; *p* = 0.333; *g* = 0.291	*t*_(52)_ = 1.48; *p* = 0.144; *g* = 0.442
Inferior temporal g. right	*t*_(52)_ = 1.62; *p* = 0.112; *g* = 0.481	*t*_(52)_ = 2.05; *p* = 0.046; *g* = 0.609
Middle temporal g. left	*t*_(52)_ = 1.15; *p* = 0.254; *g* = 0.343	*t*_(52)_ = 1.22; *p* = 0.229; *g* = 0.363
Middle temporal g. right	*t*_(52)_ = 1.12; *p* = 0.268; *g* = 0.333	*t*_(52)_ = 1.33; *p* = 0.190; *g* = 0.397
Pars opercularis of the inferior frontal g. left	*t*_(52)_ = 1.00; *p* = 0.333; *g* = 0.293	*t*_(52)_ = 0.62; *p* = 0.536; *g* = 0.185
Pars opercularis of the inferior frontal g. right	*t*_(52)_ = 0.69; *p* = 0.496; *g* = 0.204	*t*_(52)_ = 0.76; *p* = 0.451; *g* = 0.226
Superior temporal g. left	*t*_(47)_ = 0.81; *p* = 0.424; *g* = 0.195	*t*_(52)_ = 0.63; *p* = 0.533; *g* = 0.187
Superior temporal g. right	*t*_(52)_ = 1.28; *p* = 0.207; *g* = 0.379	*t*_(52)_ = 1.08; *p* = 0.286; *g* = 0.320
**(B)**		
Fusiform g. left	*t*_(52)_ = 0.44; *p* = 0.665; *g* = 0.129	*t*_(52)_ = 0.52; *p* = 0.606; *g* = 0.151
Fusiform g. right	*t*_(52)_ = 0.48; *p* = 0.636; *g* = 0.142	*t*_(52)_ = −0.52; *p* = 0.606; *g* = 0.154
Inferior parietal g. left	*t*_(52)_ = 0.26; *p* = 0.800; *g* = 0.075	*t*_(52)_ = 0.01; *p* = 0.989; *g* = 0.004
Inferior parietal g. right	*t*_(52)_ = 1.19; *p* = 0.238; *g* = 0.356	*t*_(52)_ = 0.75; *p* = 0.458; *g* = 0.223
Inferior temporal g. left	*t*_(52)_ = 0.61; *p* = 0.545; *g* = 0.180	*t*_(52)_ = 0.76; *p* = 0.452; *g* = 0.225
Inferior temporal g. right	*t*_(52)_ = 1.01; *p* = 0.320; *g* = 0.297	*t*_(52)_ = 0.69; *p* = 0.495; *g* = 0.207
Middle temporal g. left	*t*_(52)_ = 0.84; *p* = 0.405; *g* = 0.251	*t*_(52)_ = 0.72; *p* = 0.478; *g* = 0.212
Middle temporal g. right	*t*_(52)_ = 1.26; *p* = 0.214; *g* = 0.374	*t*_(52)_ = 1.44; *p* = 0.157; *g* = 0.429
Pars opercularis of the inferior frontal g. left	*t*_(52)_ = 2.11; *p* = 0.040; *g* = 0.629	*t*_(52)_ = 1.51; *p* = 0.137; *g* = 0.449
Pars opercularis of the inferior frontal g. right	*t*_(52)_ = 2.00; *p* = 0.051; *g* = 0.594	*t*_(21)_ = 0.99; *p* = 0.333; *g* = 0.342
Superior temporal g. left	*t*_(52)_ = 1.27; *p* = 0.210; *g* = 0.379	*t*_(52)_ = 0.72; *p* = 0.477; *g* = 0.212
Superior temporal g. right	*t*_(52)_ = 1.72; *p* = 0.091; *g* = 0.512	*t*_(52)_ = 1.92; *p* = 0.061; *g* = 0.571

*(A) For surface area, for each method per ROI and per hemisphere the p-value (significance at level *p* < 0.05) and the effect size (Hedges’ *g*) of reading group differences (DR vs. TR). (B) For cortical thickness, for each method per ROI and per hemisphere the p-value (significance at level *p* < 0.05) and the effect size (Hedges’ *g*) of reading group differences (DR vs. TR)*.

## Discussion

In this study, we investigated for the first time in a pediatric population whether additional manual editing of FreeSurfer data generated with the automated reconstruction pipeline is of added value. First, we investigated in 5-to-6-year-old children in six ROIs belonging to the reading network and their right counterparts if there are statistical differences in cortical thickness or surface area when generated fully automatically in FreeSurfer vs. automatically with additional manual editing. Additionally, for both morphological measures, we checked if differences between the methods are consistent across subjects. Finally, we investigated whether manual editing leads to an increased sensitivity to detect statistical differences in surface area or cortical thickness between 5-to-6-year-old children with and without dyslexia. Results revealed that the methods differ significantly from each other. Specifically, the automated method with additional manual edits reported larger and thinner ROIs than the fully automated method. Additionally, the intra-class correlations between the methods were high for all ROIs, revealing that although the methods differ significantly, the difference is consistent, and therefore leads to similar statistical inferences regarding outcome measures. Finally, effect sizes of differences in surface area or cortical thickness between children with and without dyslexia did not differ between the methods, indicating that manual editing does not lead to an increased sensitivity to detect dyslexia-related morphological brain differences in a pediatric sample, although conclusions of marginally significant findings can differ depending on the chosen method.

In line with our expectation, we observed that the fully automated method and the automated method with manual edits significantly differ in surface area and cortical thickness across ROIs. Compared to the automated method with manual edits, the fully automated method has a lower surface area and a higher cortical thickness across ROIs. Adult studies of McCarthy et al. (2015), Canna et al. (2018) and Waters et al. (2019) observed in only 0%–26.5% of the ROIs in their studies differences in brain morphometry between the methods. Also, they observed no differences in brain morphometry between the methods in ROIs of our study. Hence, it seems that pediatric data needs more manual adjustments than adult data. However, there were few methodological differences between these studies and our study. McCarthy et al. (2015) applied Bonferroni correction to 34 ROIs in their study, whereas in our study there were fewer ROIs to correct for. Additionally, whereas our study focused on fixing all possible type of edits, the study of Waters et al. (2019) focused on fixing skull stripping errors only, and the study of Canna et al. (2018) on fixing intensity normalization errors only. If we would perform a similar amount of Bonferroni corrections as McCarthy et al. (2015), i.e., correct for 34 ROIs, we would also fail to observe differences in outcome measures between the methods. Yet, suggestively their correction has been too strong. As a consequence, it cannot be confirmed that pediatric data needs more manual adaptations than adult data. Future studies should implement similar paradigms and focus more on replicating findings.

Another observation in our study was the high intra-class correlations between the methods for all ROIs, which indicate that even though the methods may differ for particular ROIs, the differences are highly consistent across subjects, and the observed relation for cortical thickness and surface area is maintained across subjects. The high intra-class correlations suggest that the choice of method is less relevant, since they indicate that both methods lead to similar statistical inferences on group estimates. Nevertheless, it remains important to stick to a single method within a study, as for certain ROIs the methods may statistically differ from each other (see also Guenette et al., 2018). In line with our results, studies of McCarthy et al. (2015) and Waters et al. (2019) observed for all brain regions high intra-class correlations between the methods. On the contrary, Guenette et al. (2018) observed high intra-class correlations for few subcortical ROIs, and, for instance, not for the amygdala and hippocampus. Past research, however, demonstrated that areas such as the amygdala and hippocampus are troublesome areas to segment for FreeSurfer and that their volumes are often overestimated (Tae et al., 2008; Nugent et al., 2013; Schoemaker et al., 2016; Schmidt et al., 2018). Hence, for these subcortical regions, extra caution is warranted.

Finally, our study revealed no significant differences between the methods in effect sizes comparing children with and without dyslexia, indicating no increased sensitivity to detect clinical group differences. Note, however, that marginally significant results can switch from significant to non-significant depending on the method used (see the right inferior temporal gyrus in Table 2A and the pars opercularis of the left inferior frontal gyrus in Table 2B), whereas stronger group differences (as observed in the bilateral area of the fusiform gyrus, Table 2A), remain significant regardless of whether manual edits are performed or not. In sum, although it might seem worthwhile to edit pediatric data, as shown by outcome differences between the methods in the segmentation process and in the output matrices that FreeSurfer generates, results indicate that the additional time and costs required to manually adjust images do not result into an increased sensitivity to detect morphological differences between the reading groups. On the contrary, one should consider whether the benefits of performing manual adaptations to optimize the data outweigh the costs of the high amount of time required for editing and the accompanying costs involved. Especially in large data sets, due to the excessive time needed for editing, benefits might not outweigh the costs, even though some quality control of the data (e.g., dealing with outliers, severe artifacts and motion) would be necessary. Hence, the results of our study and previous adult studies together indicate that manually adjusting data in FreeSurfer has a limited impact on statistical findings regarding clinical group estimates and individual neurocognitive measures. Possibly, there could be gains for pediatric images with severe motion, which had been excluded from our study. Furthermore, it should be mentioned that under certain circumstances one should always consider the adaptation of the brain image. For instance, in cases involving individual brain modeling or in cases where individual data is needed for accurate predictions of personal diagnosis or treatment outcomes. In such cases, one should aim for the most accurate result, which usually corresponds to some adaption of the image.

Currently, we have no ground truth on whether automated brain imaging methods or human experts provide the best outcomes (i.e., outcomes that are closest to the true values) regarding brain measures such as surface area or cortical thickness. For a long time, manual editing has been considered to be the golden standard, but is slowly overtaken by automated methods that get more and more accurate while constantly being adapted and improved. In the future, automatic editing procedures or machine learning techniques may become available that can assist or even replace the manual editing procedure, significantly reducing the time needed for adjustments and improving the segmentation of the brain regions. For instance, Canna et al. (2018) developed in their study an automated control point search (ACPS) that improved surface reconstructions to a similar extent as manually editing (i.e., there was a high reproducibility between the method with manual and automated edits across different data sets). Likewise, Sta Cruz et al. (2020) developed in their study a machine learning random forest imputation technique to replace missing or incorrect average values across regions by imputed values that were computed based on available multivariate information. Sta Cruz et al. (2020) tested their newly developed technique on morphological child and adult data that had been automatically processed by FreeSurfer and additionally underwent manual editing. The edited data was compared to the data acquired with the machine learning random forest imputation technique. The newly developed technique proved to be equally effective as manual editing, and would especially be interesting for studies consisting of large datasets, containing more than 250 participants.

A drawback is that we cannot make any claims regarding the necessity of additional editing for images with severe motion (*n* = 17), as they had been excluded from our analyses. Since a significant part of our study sample showed severe motion, future studies on pediatric populations are strongly encouraged to focus on diminishing motion artifacts, for instance by using prospection techniques (Brown et al., 2010; Kuperman et al., 2011; Tisdall et al., 2012). An example would be the implementation of equipment that continuously localizes and follows head position during scanning (White et al., 2010; Tisdall et al., 2012). Furthermore, we recommend implementing a template specifically designed for children in pediatric imaging studies (Alexander et al., 2017; Phan et al., 2018a). Such an atlas will lead to fewer errors in the segmentation process, and therefore more accurate outcome measures, as well as to an increased sensitivity to find clinical group differences (Phan et al., 2018a). When using an age-specific pediatric atlas as opposed to the adult atlas used by FreeSurfer, it has been shown that only half of the number of subjects is needed to detect significant morphological differences in gray matter volume between 5-to-6-year-old children with and without dyslexia. Recently developed atlases for pediatric populations are the neonatal M-CRIB atlas (Alexander et al., 2017), which is based on the Desikan-Killiany atlas, and could potentially be implemented within the FreeSurfer pipeline. Soon, it is expected that more and more age-specific pediatric atlases will become available.

To conclude, results show that although T1-weighted images fully automatically generated by FreeSurfer, and images with additional manually edits statistically differ in measures of cortical thickness and surface area in a pediatric sample of 5-to-6-year-old children with and without dyslexia, these differences are highly consistent, and additional editing does not result into an increased sensitivity to detect morphological differences between the reading groups.

## Data Availability Statement

The dataset for this manuscript is not publicly available, because the conditions of our ethics approval do not permit public archiving of anonymized study data and consent had only been obtained from participants for participation in the study and not to share data with third parties. Requests to access the dataset should be directed to Pol Ghesquière (pol.ghesquiere@kuleuven.be) explaining the purpose of their request. Following the EU general data protection regulation (GDPR), data will be released to requestors upon the following conditions: consent of the representative of the minor and a formal agreement between parties. Please note that the MRI data cannot be shared under any circumstance as MRI data are person-specific and therefore are not anonymous.

## Ethics Statement

The studies involving human participants were reviewed and approved by Ethische Commissie Onderzoek UZ/KU Leuven. Written informed consent to participate in this study was provided by the participants’ legal guardian/next of kin.

## Author Contributions

CB: conceptualization, methodology, validation, visualization, investigation, software, formal analysis, data curation, writing—original draft, writing—review and editing. TP: methodology, software, visualization, writing—review and editing. JW: methodology, validation, visualization, resources, supervision, project administration, and funding acquisition. MV: conceptualization, methodology, validation, investigation, data curation, writing—review and editing, supervision, project administration, and funding acquisition. PG: conceptualization, methodology, validation, resources, writing—review and editing, supervision, project administration, and funding acquisition.

## Conflict of Interest

Author TP was employed by the company Icometrix. The remaining authors declare that the research was conducted in the absence of any commercial or financial relationships that could be construed as a potential conflict of interest.

## Footnotes

^1^http://surfer.nmr.mgh.harvard.edu/fswiki/FsTutorial/TroubleshootingData

## References

[B2] Alexander-Bloch A. Clasen L. Stockman M. Ronan L. Lalonde F. Giedd J.. (2016). Subtle in-scanner motion biases automated measurement of brain anatomy from *in vivo* MRI. Hum. Brain Mapp. 37, 2385–2397. 10.1002/hbm.23180 27004471 PMC5110234

[B1] Alexander B. Murray A. L. Loh W. Y. Matthews L. G. Adamson C. Beare R.. (2017). A new neonatal cortical and subcortical brain atlas: the melbourne children’s regional infant brain (M-CRIB) atlas. NeuroImage 147, 841–851. 10.1016/j.neuroimage.2016.09.068 27725314

[B3] Backhausen L. L. Herting M. M. Buse J. Roessner V. Smolka M. N. Vetter N. C. (2016). Quality control of structural MRI images applied using freesurfer-a hands-on workflow to rate motion artifacts. Front. Neurosci. 10:558. 10.3389/fnins.2016.00558 27999528 PMC5138230

[B4] Barkhof F. Calabresi P. A. Miller D. H. Reingold S. C. (2009). Imaging outcomes for neuroprotection and repair in multiple sclerosis trials. Nat. Rev. Neurol. 5, 256–266. 10.1038/nrneurol.2009.41 19488083

[B5] Beelen C. Vanderauwera J. Wouters J. Vandermosten M. Ghesquière P. (2019). Atypical gray matter in children with dyslexia before the onset of reading instruction. Cortex 121, 399–413. 10.1016/j.cortex.2019.09.010 31704534

[B6] Blumenthal J. D. Zijdenbos A. Molloy E. Giedd J. N. (2002). Motion artifact in magnetic resonance imaging: implications for automated analysis. NeuroImage 16, 89–92. 10.1006/nimg.2002.1076 11969320

[B8] Brown T. T. Kuperman J. M. Erhart M. White N. S. Roddey J. C. Shankaranarayanan A.. (2010). Prospective motion correction of high-resolution magnetic resonance imaging data in children. NeuroImage 53, 139–145. 10.1016/j.neuroimage.2010.06.017 20542120 PMC3146240

[B9] Brus B. T. Voeten M. J. M. (1973). Een Minuut Test, Vorm A En B. Nijmegen, The Netherlands: Berkhout.

[B10] Canna A. Russo A. G. Ponticorvo S. Manara R. Pepino A. Sansone M.. (2018). Automated search of control points in surface-based morphometry. NeuroImage 176, 56–70. 10.1016/j.neuroimage.2018.04.035 29673966

[B11] Cardinale F. Chinnici G. Bramerio M. Mai R. Sartori I. Cossu M.. (2014). Validation of freesurfer-estimated brain cortical thickness: comparison with histologic measurements. Neuroinformatics 12, 535–542. 10.1007/s12021-014-9229-2 24789776

[B12] Dale A. M. Fischl B. Sereno M. I. (1999). Cortical surface-based analysis. I. segmentation and surface reconstruction. NeuroImage 9, 179–194. 10.1006/nimg.1998.0395 9931268

[B13] Dale A. M. Sereno M. I. (1993). Improved localization of cortical activity by combining EEG and MEG with MRI cortical surface reconstruction: a linear approach. J. Cogn. Neurosci. 5, 162–176. 10.1162/jocn.1993.5.2.162 23972151

[B14] Desikan R. S. Ségonne F. Fischl B. Quinn B. T. Dickerson B. C. Blacker D.. (2006). An automated labeling system for subdividing the human cerebral cortex on MRI scans into gyral based regions of interest. NeuroImage 31, 968–980. 10.1016/j.neuroimage.2006.01.021 16530430

[B15] Destrieux C. Fischl B. Dale A. Halgren E. (2010). Automatic parcellation of human cortical gyri and sulci using standard anatomical nomenclature. NeuroImage 53, 1–15. 10.1016/j.neuroimage.2010.06.010 20547229 PMC2937159

[B17] Durston S. Hulshoff Pol H. E. Casey B. J. Giedd J. N. Buitelaar J. K. van Engeland H. (2001). Anatomical MRI of the developing human brain: what have we learned? J. Am. Acad. Child Adolesc. Psychiatry 40, 1012–1020. 10.1097/00004583-200109000-00009 11556624

[B18] Eickhoff S. B. Thirion B. Varoquaux G. Bzdok D. (2015). Connectivity-based parcellation: critique and implications. Hum. Brain Mapp. 36, 4771–4792. 10.1002/hbm.22933 26409749 PMC6869530

[B19] Estes A. Munson J. Rogers S. J. Greenson J. Winter J. Dawson G. (2015). Long-term outcomes of early intervention in 6-year-old children with autism spectrum disorder. J. Am. Acad. Child Adolesc. Psychiatry 54, 580–587. 10.1016/j.jaac.2015.04.005 26088663 PMC4475272

[B20] Fallucca E. MacMaster F. P. Haddad J. Easter P. Dick R. May G.. (2011). Distinguishing between major depressive disorder and obsessive-compulsive disorder in children by measuring regional cortical thickness. Arch. Gen. Psychiatry 68, 527–533. 10.1001/archgenpsychiatry.2011.36 21536980 PMC4777351

[B21] Fischl B. Dale A. M. (2000). Measuring the thickness of the human cerebral cortex from magnetic resonance images. Proc. Natl. Acad. Sci. U S A 97, 11050–11055. 10.1073/pnas.200033797 10984517 PMC27146

[B22] Fischl B. Liu A. Dale A. M. (2001). Automated manifold surgery: constructing geometrically accurate and topologically correct models of the human cerebral cortex. IEEE Trans. Med. Imaging 20, 70–80. 10.1109/42.906426 11293693

[B24] Fischl B. Salat D. H. Busa E. Albert M. Dieterich M. Haselgrove C.. (2002). Whole brain segmentation: automated labeling of neuroanatomical structures in the human brain. Neuron 33, 341–355. 10.1016/s0896-6273(02)00569-x 11832223

[B23] Fischl B. Salat D. H. van der Kouwe A. J. W. Makris N. Ségonne F. Quinn B. T.. (2004). Sequence-independent segmentation of magnetic resonance images. NeuroImage 23, 69–84. 10.1016/j.neuroimage.2004.07.016 15501102

[B25] Fischl B. Sereno M. I. Dale A. M. (1999a). Cortical surface-based analysis. II: inflation, flattening and a surface-based coordinate system. NeuroImage 9, 195–207. 10.1006/nimg.1998.0396 9931269

[B26] Fischl B. Sereno M. I. Tootell R. B. H. Dale A. M. (1999b). High-resolution intersubject averaging and a coordinate system for the cortical surface. Hum. Brain Mapp. 8, 272–284. 10.1002/(sici)1097-0193(1999)8:4<272::aid-hbm10>3.0.co;2-4 10619420 PMC6873338

[B27] Gedamu E. L. Collins D. L. Arnold D. L. (2008). Automated quality control of brain MR images. J. Magn. Reson. Imaging 28, 308–319. 10.1002/jmri.21434 18666143

[B28] Gold A. L. Steuber E. R. White L. K. Pacheco J. Sachs J. F. Pagliaccio D.. (2017). Cortical thickness and subcortical gray matter volume in pediatric anxiety disorders. Neuropsychopharmacology 42, 2423–2433. 10.1038/npp.2017.83 28436445 PMC5645752

[B29] Guenette J. P. Stern R. A. Tripodis Y. Chua A. S. Schultz V. Sydnor V. J.. (2018). Automated versus manual segmentation of brain region volumes in former football players. Neuroimage Clin. 18, 888–896. 10.1016/j.nicl.2018.03.026 29876273 PMC5988230

[B30] Hedman A. M. van Haren N. E. M. Schnack H. G. Kahn R. S. Hulshoff Pol H. E. (2012). Human brain changes across the life span: a review of 56 longitudinal magnetic resonance imaging studies. Hum. Brain Mapp. 33, 1987–2002. 10.1002/hbm.21334 21915942 PMC6870052

[B31] Jones K. Daley D. Hutchings J. Bywater T. Eames C. (2007). Efficacy of the incredible years basic parent training programme as an early intervention for children with conduct problems and ADHD. Child Care Health Dev. 33, 749–756. 10.1111/j.1365-2214.2007.00747.x 17944785

[B32] Kapellou O. Counsell S. J. Kennea N. Dyet L. Saeed N. Stark J.. (2006). Abnormal cortical development after premature birth shown by altered allometric scaling of brain growth. PLoS Med. 3:e265. 10.1371/journal.pmed.0030265 16866579 PMC1523379

[B33] Kuperman J. M. Brown T. T. Ahmadi M. E. Erhart M. J. White N. S. Roddey A.. (2011). Prospective motion correction improves diagnostic utility of pediatric MRI scans. Pediatr. Radiol. 41, 1578–1582. 10.1007/s00247-011-2205-1 21779892 PMC3933373

[B34] Lovett M. W. Frijters J. C. Wolf M. Steinbach K. A. Sevcik R. A. Morris R. D. (2017). Early intervention for children at risk for reading disabilities: the impact of grade at intervention and individual differences on intervention outcomes. J. Educ. Psychol. 109, 889–914. 10.1037/edu0000181 PMC9164258 35664550

[B35] Mahajan R. Dirlikov B. Crocetti D. Mostofsky S. H. (2016). Motor circuit anatomy in children with autism spectrum disorder with or without attention deficit hyperactivity disorder. Med. Hypotheses 9, 67–81. 10.1002/aur.1497 25962921 PMC5412258

[B36] Makowski C. Béland S. Kostopoulos P. Bhagwat N. Devenyi G. A. Malla A. K.. (2018). Evaluating accuracy of striatal, pallidal, and thalamic segmentation methods: comparing automated approaches to manual delineation. NeuroImage 170, 182–198. 10.1016/j.neuroimage.2017.02.069 28259781

[B37] Makrygianni M. K. Reed P. (2010). A meta-analytic review of the effectiveness of behavioural early intervention programs for children with autistic spectrum disorders. Res. Autism Spectr. Disord. 4, 577–593. 10.1016/j.rasd.2010.01.014

[B38] Mayer A. R. Hanlon F. M. Ling J. M. (2015). Gray matter abnormalities in pediatric mild traumatic brain injury. J. Neurotrauma 32, 723–730. 10.1089/neu.2014.3534 25313896

[B39] McCarthy C. S. Ramprashad A. Thompson C. Botti J. A. Coman I. L. Kates W. R. (2015). A comparison of freesurfer-generated data with and without manual intervention. Front. Neurosci. 9:379. 10.3389/fnins.2015.00379 26539075 PMC4612506

[B40] Merkley T. L. Bigler E. D. Wilde E. A. McCauley S. R. Hunter J. V. Levin H. S. (2008). Short communication: diffuse changes in cortical thickness in pediatric moderate-to-severe traumatic brain injury. J. Neurotrauma 25, 1343–1345. 10.1089/neu.2008.0615 19061377 PMC2747789

[B41] Muzik O. Chugani D. C. Juhász C. Shen C. Chugani H. T. (2000). Statistical parametric mapping: assessment of application in children. NeuroImage 12, 538–549. 10.1006/nimg.2000.0651 11034861

[B43] Nugent T. F. III. Herman D. H. Ordonez A. Greenstein D. Hayashi K. M. Lenane M.. (2007). Dynamic mapping of hippocampal development in childhood onset schizophrenia. Schizophr. Res. 90, 62–70. 10.1016/j.schres.2006.10.014 17161938

[B42] Nugent A. C. Luckenbaugh D. A. Wood S. E. Bogers W. Zarate C. A. Drevets W. C. (2013). Automated subcortical segmentation using FIRST: test-retest reliability, interscanner reliability, and comparison to manual segmentation. Hum. Brain Mapp. 34, 2313–2329. 10.1002/hbm.22068 22815187 PMC3479333

[B44] Overvliet G. M. Besseling R. M. H. Jansen J. F. A. van der Kruijs S. J. M. Vles J. S. H. Hofman P. A. M.. (2013). Early onset of cortical thinning in children with rolandic epilepsy. Neuroimage Clin. 2, 434–439. 10.1016/j.nicl.2013.03.008 24179797 PMC3777705

[B45] Ozernov-Palchik O. Gaab N. (2016). Tackling the ‘Dyslexia Paradox’: reading brain and behavior for early markers of developmental dyslexia. Wiley Interdiscip. Rev. Cogn. Sci. 7, 156–176. 10.1002/wcs.1383 26836227 PMC4761294

[B46] Panizzon M. S. Fennema-Notestine C. Eyler L. T. Jernigan T. L. Prom-Wormley E. Neale M.. (2009). Distinct genetic influences on cortical surface area and cortical thickness. Cereb. Cortex 19, 2728–2735. 10.1093/cercor/bhp026 19299253 PMC2758684

[B47] Peters-Scheffer N. Didden R. Korzilius H. Sturmey P. (2011). A meta-analytic study on the effectiveness of comprehensive ABA-based early intervention programs for children with autism spectrum disorders. Res. Autism Spectr. Disord. 5, 60–69. 10.1016/j.rasd.2010.03.011

[B48] Phan T. V. Sima D. M. Beelen C. Vanderauwera J. Smeets D. Vandermosten M. (2018a). Evaluation of methods for volumetric analysis of pediatric brain data: the childmetrix pipeline versus adult-based approaches. Neuroimage Clin. 19, 734–744. 10.1016/j.nicl.2018.05.030 30003026 PMC6040578

[B49] Phan T. V. Smeets D. Talcott J. B. Vandermosten M. (2018b). Processing of structural neuroimaging data in young children: bridging the gap between current practice and state-of-the-art methods. Dev. Cogn. Neurosci. 33, 206–223. 10.1016/j.dcn.2017.08.009 29033222 PMC6969273

[B50] Reuter M. Tisdall M. D. Qureshi A. Buckner R. L. van der Kouwe A. J. W. Fischl B. (2015). Head motion during MRI acquisition reduces gray matter volume and thickness estimates. NeuroImage 107, 107–115. 10.1016/j.neuroimage.2014.12.006 25498430 PMC4300248

[B51] Richlan F. Kronbichler M. Wimmer H. (2009). Functional abnormalities in the dyslexic brain: a quantitative meta-analysis of neuroimaging studies. Hum. Brain Mapp. 30, 3299–3308. 10.1002/hbm.20752 19288465 PMC2989182

[B52] Rosas H. D. Liu A. K. Hersch S. Glessner M. Ferrante R. J. Salat D. H.. (2002). Regional and progressive thinning of the cortical ribbon in Huntington’s disease. Neurology 58, 695–701. 10.1212/wnl.58.5.695 11889230

[B53] Rosas H. D. Reuter M. Doros G. Lee S. Y. Triggs T. Malarick K.. (2011). A tale of two factors: what determines the rate of progression in Huntington’s disease? A longitudinal MRI study. Mov. Disord. 26, 1691–1697. 10.1002/mds.23762 21611979 PMC3155608

[B54] Schmidt M. F. Storrs J. M. Freeman K. B. Jack C. R. Jr. Turner S. T. Griswold M. E.. (2018). A comparison of manual tracing and freesurfer for estimating hippocampal volume over the adult lifespan. Hum. Brain Mapp. 39, 2500–2513. 10.1002/hbm.24017 29468773 PMC5951757

[B55] Schoemaker D. Buss C. Head K. Sandman C. A. Davis E. P. Chakravarty M. M.. (2016). Hippocampus and amygdala volumes from magnetic resonance images in children: assessing accuracy of FreeSurfer and FSL against manual segmentation. NeuroImage 129, 1–14. 10.1016/j.neuroimage.2016.01.038 26824403 PMC7243960

[B56] Ségonne F. Dale A. M. Busa E. Glessner M. Salat D. Hahn H. K.. (2004). A hybrid approach to the skull stripping problem in MRI. NeuroImage 22, 1060–1075. 10.1016/j.neuroimage.2004.03.032 15219578

[B57] Ségonne F. Pacheco J. Fischl B. (2007). Geometrically accurate topology-correction of cortical surfaces using nonseparating loops. IEEE Trans. Med. Imaging 26, 518–529. 10.1109/tmi.2006.887364 17427739

[B58] Sied J. G. Zijdenbos A. P. Evans A. C. (1998). A nonparametric method for automatic correction of intensity nonuniformity in MRI data. IEEE Trans. Med. Imaging 17, 87–97. 10.1109/42.668698 9617910

[B59] Spinks R. Magnotta V. A. Andreasen N. C. Albright K. C. Ziebell S. Nopoulos P.. (2002). Manual and automated measurement of the whole thalamus and mediodorsal nucleus using magnetic resonance imaging. NeuroImage 17, 631–642. 10.1006/nimg.2002.1185 12377139

[B60] Sta Cruz S. Dinov I. D. Herting M. M. González-Zacarías C. Kim H. Toga A. W.. (2020). Imputation strategy for reliable regional MRI morphological measurements. Neuroinformatics 18, 59–70. 10.1007/s12021-019-09426-x 31054076 PMC6829024

[B61] Tae W. S. Kim S. S. Lee K. U. Nam E. C. Kim K. W. (2008). Validation of hippocampal volumes measured using a manual method and two automated methods (FreeSurfer and IBASPM) in chronic major depressive disorder. Neuroradiology 50, 569–581. 10.1007/s00234-008-0383-9 18414838

[B62] Theys C. Wouters J. Ghesquière P. (2014). Diffusion tensor imaging and resting-state functional MRI-scanning in 5- and 6-year-old children: training protocol and motion assessment. PLoS One 9:e94019. 10.1371/journal.pone.0094019 24718364 PMC3981727

[B63] Tisdall M. D. Hess T. A. Reuter M. Meintjes E. M. Fischl B. van der Kouwe A. J. W. (2012). Volumetric navigators (VNavs) for prospective motion correction and selective reacquisition in neuroanatomical MRI. Magn. Reson. Med. 68, 389–399. 10.1002/mrm.23228 22213578 PMC3320676

[B7] van den Bos K. P. Spelberg H. C. Scheepstra A. J. M. de Vries J. R. (1994). De Klepel Vorm A En B: Verantwoording, Handleiding, Diagnostiek En Behandeling. Nijmegen: Berkhout.

[B16] van Dijk K. R. A. Sabuncu M. R. Buckner R. L. (2012). The influence of head motion on intrinsic functional connectivity MRI. NeuroImage 59, 431–438. 10.1016/j.neuroimage.2011.07.044 21810475 PMC3683830

[B64] Vanvooren S. Poelmans H. Hofmann M. Ghesquière P. Wouters J. (2014). Hemispheric asymmetry in auditory processing of speech envelope modulations in prereading children. J. Neurosci. 34, 1523–1529. 10.1523/JNEUROSCI.3209-13.2014 24453339 PMC6705306

[B65] Wanzek J. Vaughn S. (2007). Research-based implications from extensive early reading interventions. School Psychol. Rev. 36, 541–561. 10.1080/02796015.2007.12087917

[B66] Waters A. B. Mace R. A. Sawyer K. S. Gansler D. A. (2019). Identifying errors in freesurfer automated skull stripping and the incremental utility of manual intervention. Brain Imaging Behav. 13, 1281–1291. 10.1007/s11682-018-9951-8 30145718

[B68] White T. Jansen P. R. Muetzel R. L. Sudre G. El Marroun H. Tiemeier H.. (2018). Automated quality assessment of structural magnetic resonance images in children: comparison with visual inspection and surface-based reconstruction. Hum. Brain Mapp. 39, 1218–1231. 10.1002/hbm.23911 29206318 PMC6866370

[B67] White N. Roddey C. Shankaranarayanan A. Han E. Rettmann D. Santos J.. (2010). PROMO: real-time prospective motion correction in MRI using image-based tracking. Magn. Reson. Med. 63, 91–105. 10.1002/mrm.22176 20027635 PMC2892665

[B69] Widjaja E. Mahmoodabadi S. Z. Snead O. C. III. Almehdar A. Smith M. L. (2011). Widespread cortical thinning in children with frontal lobe epilepsy. Epilepsia 52, 1685–1691. 10.1111/j.1528-1167.2011.03085.x 21627647

[B70] Winkler A. M. Kochunov P. Blangero J. Almasy L. Zilles K. Fox P. T.. (2010). Cortical thickness or grey matter volume? The importance of selecting the phenotype for imaging genetics studies. NeuroImage 53, 1135–1146. 10.1016/j.neuroimage.2009.12.028 20006715 PMC2891595

[B71] Wolosin S. M. Richardson M. E. Hennessey J. G. Denckla M. B. Mostofsky S. H. (2009). Abnormal cerebral cortex structure in children with ADHD. Hum. Brain Mapp. 30, 175–184. 10.1002/hbm.20496 17985349 PMC2883170

[B72] Wozniak J. R. Mueller B. A. Bell C. J. Muetzel R. L. Hoecker H. L. Boys C. J.. (2013). Global functional connectivity abnormalities in children with fetal alcohol spectrum disorders. Alcohol. Clin. Exp. Res. 37, 748–756. 10.1111/acer.12024 23240997 PMC3610852

[B73] Yang D. Y. J. Beam D. Pelphrey K. A. Abdullahi S. Jou R. J. (2016). Cortical morphological markers in children with autism: a structural magnetic resonance imaging study of thickness, area, volume, and gyrification. Mol. Autism 7:11. 10.1186/s13229-016-0076-x 26816612 PMC4727390

[B74] Yoon U. Fonov V. S. Perusse D. Evans A. C. (2009). The effect of template choice on morphometric analysis of pediatric brain data. NeuroImage 45, 769–777. 10.1016/j.neuroimage.2008.12.046 19167509

