# Supplementary material for: Investigating the Added Value of FreeSurfer’s Manual Editing Procedure for the Study of the Reading Network in a Pediatric Population
Source: Front Hum Neurosci. 2020 Apr 24;14:143. doi: 10.3389/fnhum.2020.00143 (PMC7194167; doi:10.3389/fnhum.2020.00143)
Supplement: Supplementary file 1 [file Data_Sheet_1.PDF]

Supplementary Material

1 FreeSurfer Manual Editing Protocol

Manual Editing in FreeSurfer consists of **5 different steps**:

- Fixing Skull Stripping Errors
- Fixing Intensity Normalization Errors
- Fixing Topological Errors
- Fixing White Matter Errors
- Fixing Pial Errors

One and the same processing step, i.e. "white matter segmentation"

Manual editing steps should be followed **in fixed order**, in line with the **Automated Reconstruction Processing Stream Pipeline** of FreeSurfer. The higher up in the processing stream, the earlier a fixing step can be performed.

In the **automated reconstruction processing stream pipeline** of FreeSurfer, a T1-weighted image undergoes in the following order:

1. Skull stripping → skull stripping errors can be fixed
2. Volumetric labeling
3. Intensity normalization → intensity normalization errors can be fixed
4. White matter segmentation → white matter segmentation errors (i.e. topological- and white matter errors) can be fixed
5. Surface atlas registration
6. Surface extraction → pial errors can be fixed
7. Gyral labeling

Edits are performed in **Freeview**, the supporting toolbox of FreeSurfer. First, in the **command-line window** a path to the FreeSurfer directory needs to be set. In the **FreeSurfer directory**, the following command should be written in order to load a brain image in Freeview:

freeview -v \
<subject name>/mri/T1.mgz \
<subject name>/mri/wm.mgz \
<subject name>/mri/brainmask.mgz \
<subject name>/mri/aseg.mgz:opacity=0.2 \
-f <subject name>/surf/lh.white:edgecolor=blue \
<subject name>/surf/lh.pial:edgecolor=red \
<subject name>/surf/rh.white:edgecolor=blue \
<subject name>/surf/rh.pial:edgecolor=red \

➔ **Freeview opens** and the image is loaded. The image can now be checked for errors and edited manually.

In Freeview, T1-images can undergo manual edits. These edits need to be performed in **at least two** of the three brain views (i.e. coronal, sagittal and axial). In addition, inflated and meshed 3D surfaces should be checked for topological errors.

Use the mouse buttons and -scroll, and the arrows of the keyboard to zoom-in/zoom-out the brain image, change the position of the brain image as well as to go through brain slices from top-to-bottom (or vice versa) and from left-to-right (or vice versa).

The manual editing steps are described in detail below.

Step 1. Fixing Skull Stripping Errors

Before editing:

- Highlight in the left menu under the tab '**Volume':** "**Brainmask"** and **"T1"** (deselect the others).
- Double-click with the mouse to put **"Brainmask"** on top (so that edits will be performed in the brainmask).
- Use **Alt + V** to toggle between "**Brainmask"** and **"T1"** (to better identify where the errors are).
- Above the image, the coronal, sagittal or axial view can be selected. Editing the **sagittal view** is recommended, since in this view skull stripping errors can be seen best.

There are **3** options to **fix skull stripping errors**:

1. Adjust watershed threshold (recommended)
2. Clone-in missing voxels
3. Use a g-cut flag (only when too much skull is left behind)

1. Adjusting watershed threshold

The watershed threshold can be adjusted by using a formula, which adapts the **'h'** (i.e. 'preflooding height'). The standard value of the "h' is 25. If **too much skull** is removed, you should **lower the h,** and if **too little skull** is removed, you should **heighten the h**:

- h of 0-15: if too much skull is removed; part of the brain is missing
- h of 35-50: if too little skull is removed; part of the skull is left behind

Close Freeview. Write the following formula in the command-line window in order to adapt the 'h': **recon-all –skullstrip -wsthresh <h> -clean-bm -subjid <subject name>** (for the 'h' any number between 0 and 50, either above or below 25, can be chosen). Load the image again and check if the skull stripping errors have disappeared. Otherwise, try option 2 or 3.

2. **Clone-in missing voxels**

Voxels can be cloned from **“T1”** to **“Brainmask”** using the **editing tool** (tool with the pencil, third from left at the top) in Freeview. A voxel edit screen appears and the tool with the blue and white squared boxes should to be selected. A **brush size** of 1 or higher is needed to clone-in the missing voxels. The **reference** should be the T1-image and the editing will be done in Brainmask. With the backward arrow or the freehand tool, mistakes in cloning can be made undone (shift + left mouse click). Click on **‘save volume’** after all the missing voxels are cloned-in.

3. **Use a g-cut flag**

A g-cut flag is only used when **a small part of dura** is left behind. Close Freeview. Write the following formula in the command-line to apply a g-cut: **recon-all -skullstrip -clean-bm -gcut -subjid <subject name>**. Overlay **“brainmask.gcuts.mgz”** (which is created via the formula) on **“T1”** to see whether the g-cut process worked successfully. Use this newly created brainmask-file instead of the original one (deselect) to perform additional edits in, if needed.

***Important*:** edits always need to be **saved**. Click on **save** after having performed option 1, 2 and/or 3. In addition, images can be processed further from this point onwards. Write the following formula in the command-line to restart the automated processing of the image after having fixed skull stripping errors: **recon-all –autorecon2 –autorecon3 subjid <subject name>**.

Step 2. Fixing Intensity Normalization Errors

To fix intensity normalization errors, the adding of **control points** will be needed. Control points are implemented in order to increase the white matter surface. They heighten the intensity of areas at the border of the white matter surface that are too low to be considered white matter (i.e. T1-value is below 80), but of which the experimenter visually observes that they are not gray matter and belong to the white matter surface (i.e. T1-value of these areas should be 110).

Open Freeview. In the left menu, highlight **Brainmask** and **T1.** Select **Brainmask** on top.
Control points look like **small green dots**. They should be used **sparsely** and placed **in middle parts of the areas** that are intended to become parts of the white matter surface. Add control points for **T1-values: 80-110** (the T1-values can be found below the picture). Lower values than 80 cannot be considered as white matter.

Go to:
File → new point set

Enter:
‘control.dat’

***Important*: Save** ‘control.dat’ in the **tmp folder of the subject** (otherwise adjustments will *not* be taken into account)**!**

→ To rerun step 2:
recon-all -autorecon2-cp -autorecon3 -subjid <subject name>

Step 3. Fixing Topological Errors

Open Freeview. Select **“brainmask”** and **“WM”** in the left menu, and select **“WM”** on top.
Via the sidebar in the left menu, the colour of the WM-mask can be adjusted. A recommended colour for fixing topological errors is pink/orange. Therefore, select **Heath-map** in the left menu.

To fix the errors, click in the top menu on **3D-vision**. After that, click in the left menu on the **‘surface’** button and select **‘surface and mesh’** under the **‘Render’** drop down menu. A meshed 3D surface will appear. Check for **holes** in the meshed 3D surface. Then, go back to the **2D-vision** by deselecting the 3D-vision and add voxels at the corresponding parts where holes were visible in the 3d-vision. For adding voxels, use the **recon-edit** icon in the top left menu with a brush value of 255. Repeat this process, if needed. **Save** the edits by clicking on the save icon.

Step 4. Fixing White Matter Errors

Open Freeview. Select **“brainmask”** and **“WM”** in the left menu, and select **“WM”** on top. Via the sidebar in the left menu, the colour of the WM mask can be adjusted. A recommended colour to make fixing errors easier is pink/orange. Therefore, select **Heath-map** in the left menu. Click on the **recon-edit** icon**.** Use a brush value of 255 to add voxels and an eraser (hold the shift-button) to delete voxels. Voxels should be **added** at locations where it is clear that **WM is underneath**, although this is ***not*** visible on the WM-mask (i.e. areas are not selected as part of WM, whereas they should be part of WM). Voxels should be **deleted** at locations where it is clear that **no WM is underneath**, although this is visible on the WM mask (i.e. areas are selected as part of WM, whereas they should not be part of WM).

Save the edits by clicking on the save icon.

➔ To rerun step **3** or **4**:
recon-all -autorecon2-wm -autorecon3 -subjid <subject name>

Step 5. Fixing Pial Errors

Open Freeview. Select **“brainmask”** and **“T1”** in the left menu, and select **“Brainmask”** on top. Click on the **recon-edit** icon. Use a brush value of **255** to add voxels and an eraser (hold the shift-button) to delete voxels. Voxels should be added or deleted at locations where it is visible that the pial (red) line does not follow the gm-csf border. Voxels should be **added** at locations where it is visible that the pial line **cuts into the brain.** Voxels should be deleted at locations where it is visible that the pial line **exceeds the skull**. Do ***not*** edit the cerebellum!

➔ To rerun step 5:
recon-all -autorecon-pial -subjid <subject name>

Important: Whenever edits are performed **across multiple processing stages,** the following command is recommended to be used:
recon-all -all -subjid <subject name>
